# Supplementary material for: Unlocking the potentials of Ustilago trichophora for up‐cycling polyurethane‐derived monomer 1,4‐butanediol
Source: Microb Biotechnol. 2024 Mar 7;17(3):e14384. doi: 10.1111/1751-7915.14384 (PMC10920939; doi:10.1111/1751-7915.14384)
Supplement: Supplementary file 1 — Data S1. [file MBT2-17-e14384-s001.pdf]

Unlocking the potentials of *Ustilago trichophora* for up-cycling polyurethane-derived monomer 1,4-butanediol

An N.T. Phan*, Lisa Prigolovkin, Lars M. Blank

Institute of Applied Microbiology - iAMB, Aachen Biology and Biotechnology – ABBt, RWTH Aachen University, Worringerweg 1, 52074 Aachen, Germany.

Supplemental Experimental procedures

IELC method validation

Samples were analyzed on three different days in triplicate. The RSD values was calculated by dividing the standard deviation by the measured concentration. The percent accuracy was determined by $[(C_m – C_k)/C_k] * 100\%$, where C_m is the measured concentration and C_k is the known concentration of the analytes in standard mixtures. The method was considered validated if the RSD and accuracy were within 15% of the measured and known concentrations for both the intra-day and the inter-day results.

Supplemental figures

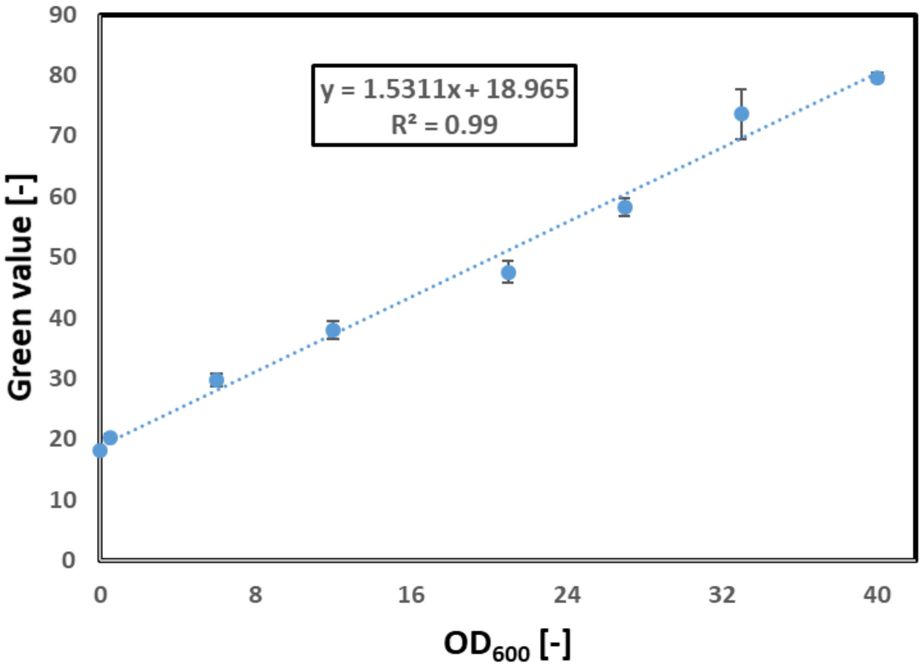

Figure S1. Correlation between green value and OD_{600} for *U. trichophora*. *U. trichophora* TZ1 was cultivated with MES-MTM with 50 g/L glycerol as carbon source. Cells were collected after 72h and used to prepare solution with the pre-defined OD_{600} ranging from 0.5 to 40. The sample for OD_{600} of 0 was the medium MES-MTM with 50 g/L glycerol. All solutions with pre-defined OD_{600} were loaded into 24-deep-well microplates and measured the green value by Growth Profiler GP960 (*EnzyScreen*, The Netherlands). Experiments were performed with three biological replicates. The error bars indicate the standard deviation.

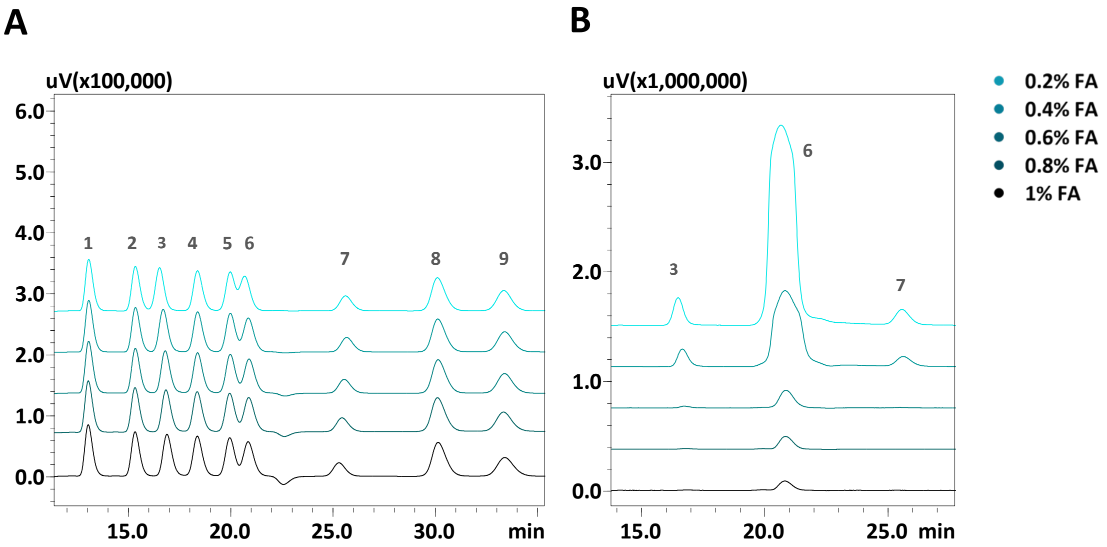

Figure S2. Effects of FA concentration. (A) RI chromatograms. (B) UV chromatograms. The standard mixtures contained (1) cellobiose; (2) glucose; (3) quinic acid; (4) xylitol; (5) glycolaldehyde; (6) itaconic acid; (7) adipic acid; (8) MeOH, and (9) BDO. Experiments were performed with a FA concentration from 0.2% to 1%, a flow rate of 0.4 mL/min, a column temperature of 40 °C, and without organic modifiers.

Supplemental tables

Table S1: Method validation

	C_k (g/L)	Intra-day			Inter-day		
		**C_m (g/L)**	**RSD (%)**	**Accuracy (%)**	**C_m (g/L)**	**RSD (%)**	**Accuracy (%)**
**Cellobiose**	10	9,8	0.3	98	10.4	8	104
**Glucose**	10	9.8	0.6	98	10.3	7	103
**Quinic acid**	10	9.8	0.6	98	10.3	7	103
**Xylitol**	10	9.9	0.8	99	10.3	6	103
**Glycolaldehyde**	10	9.9	0.7	99	10.3	5	103
**Itaconic acid**	10	9.9	1.2	99	10.3	6	103
**Adipic acid**	5	4.8	2.5	95	4.9	10	98
**MeOH**	10	9.5	1.8	95	10.1	7	101
**BDO**	10	10.0	1.3	100	10.3	6	103

C_k: the known concentration of the analytes.
C_m: the measured concentration of the analytes.

Table S2: UV/RI detectors

Compound ID	Ret. Time	Quantification		
		Range (g/L)	Equation	R^2
**2-ketogluconic acid**	12.49	0.01 - 20	y = 323004x + 84916	0.99
**Acetic acid**	23.93	0.04 - 20	y = 318966x + 38214	1
**Aconic acid**	18.80	0.08 - 20	y = 108960x - 2751.5	1
**Adipic acid**	25.36	0.01 - 5	y = 372380x + 27356	0.99
**Alpha-ketoglutaric acid**	12.97	0.001 - 5	y = 3E+06x + 170251	0.99
**Arabinose**	16.94	0.08 - 10	y = 104636x - 18204	1
**BDO**	32.84	0.01 - 10	y = 74267x - 6291.1	1
**Cellobiose**	12.57	0.005 - 20	y = 112227x + 1472.8	1
**EG**	25.03	0.01 - 20	y = 66379x - 2159.4	1
**Erythritol**	18.61	0.02 - 20	y = 101941x + 383.53	1
**Fructose**	15.96	0.02 - 10	y = 122359x - 15038	1
**Fumaric acid**	21.34	0.02 - 2.5	y = 1E+06x - 37658	1
**Galactose**	15.41	0.02 - 20	y = 98131x + 2788.4	1
**Gluconic acid**	14.48	0.08 - 10	y = 251095x - 19919	1
**Glucose**	14.74	0.01 - 20	y = 114349x - 1628.9	1
**Glycerol**	21.10	0.08 - 20	y = 85957x + 12783	1
**Glycolic acid**	19.30	0.001 - 10	y = 2E+06x + 263644	0.99
**Glyoxylic acid**	15.20	0.001 - 2.5	y = 6E+06x + 106118	1
**Inositol**	15.27	0.02 - 20	y = 134266x - 6021.2	1
**Iso/Citric acid**	13.29	0.02 - 20	y = 497812x + 116819	1
**Itaconic acid**	20.27	0.02- 20	y = 2E+07x + 191481	1
**Lactose**	12.86	0.02 - 10	y = 110661x - 14488	1
**Malic acid**	15.35	0.08 - 10	y = 530774x - 57569	1
**Maltose**	12.60	0.01 - 20	y = 155198x - 3035.6	1
**Mannitol**	16.32	0.01 - 5	y = 125447x - 1756.8	1
**Mannose**	15.50	0.01 - 20	y = 123858x - 1377.8	0.99
**Propionic acid**	27.82	0.08 - 20	y = 271649x - 69225	0.99
**Quinic acid**	15.97	0.02 - 20	y = 273469x - 6796.8	1
**Rhamnose**	16.32	0.02 - 20	y = 103262x + 1713.4	1
**Ribose**	17.41	0.02 - 20	y = 94413x - 1881.9	0.99
**Sorbitol**	16.50	0.02 - 20	y = 114021x - 7956.8	1
**Sorbose**	15.02	0.01 - 20	y = 115937x + 104.75	0.98
**Succinic acid**	18.89	0.01 - 2.5	y = 1E+07x + 272309	0.99

**Sucrose**	12.66	0.02 - 20	y = 57830x - 4731.2	1
**Tartaric acid**	13.88	0.01 - 20	y = 724911x + 83746	0.99
**Trehalose**	12.55	0.005 - 20	y = 207201x + 2227	1
**Xylitol**	17.76	0.01 - 20	y = 135076x - 2438.7	1
**Xylose**	15.59	0.02 - 20	y = 88764x - 3042.3	1

Table S3: Multiple Reaction Monitoring Library for MS/MS

Compound ID	Ret. Time	Adduct ion	Polarity	Quantification ion			Reference ion 1			Reference ion 2			Quantification		
				Precursor ion	Product ion	CE (V)	Precursor ion	Product ion	CE (V)	Precursor ion	Product ion	CE (V)	Range (g/L)	Equation	R^2
2-ketogluconic acid	12.49	[M-H]-	(-)	193	103	12	193	59	21	193	89	13	0.005 - 1.25	y = 8E+06x - 74634	1
Acetic acid	23.93	[M+H]+	(+)	61	43	-10	61	41	-11	61	44	-18	0.005 - 20	y = 779089x - 6696.4	1
Aconic acid	18.80	[M-H]-	(-)	173	111	10	173	129	11	173	111	10	0.04 - 5	y = 87183x + 18055	0.99
Adipic acid	25.36	[M-H]-	(-)	145	101	14	145	83	14	145	81	21	0.02 - 5	y = 2E+06x - 260622	1
Alpha-ketoglutaric acid	12.97	[M+H]+	(+)	147	129	-12	147	97	-15	147	56	-26	0.02 - 5	y = 1E+06x + 24945	1
Arabinose	16.94	[M+HCOO-]-	(-)	195	89	12	195	149	10	195	59	22	0.01 - 5	y = 238547x + 19731	0.99
BDO	32.84	[M+H]+	(+)	91	43	-17	91	55	-12	91	73	-9	0.01 - 2.5	y = 4E+07x + 882321	1
Cellobiose	12.57	[M+HCOO-]-	(-)	387	341	8	387	161	12	387	101	22	0.01 - 1.25	y = 159520x - 8273	1
EG	25.03	[M+H]+	(+)	63	45	-10	63	43	-23	63	27	-24	0.01 - 1.25	y = 4E+06x + 156809	0.99
Erythritol	18.61	[M+H]+	(+)	123	69	-12	123	105	-10	123	87	-10	0.001 - 10	y = 1E+07x - 70125	1
Fructose	15.96	[M-H]-	(-)	179	89	8	179	59	17	179	135	14	0.08 - 5	y = 15645x + 5972.6	0.99
Fumaric acid	21.34	[M-H]-	(-)	115	71	10	115	98	22				0.02 - 2.5	y = 194879x - 12341	0.99
Galactose	15.41	[M-H]-	(-)	179	89	8	179	135	14	179	59	18	0.02 - 1.25	y = 1541.2x + 2213.1	1
Gluconic acid	14.48	[M-H]-	(-)	195	129	13	195	75	17	195	99	14	0.04 - 10	y = 686420x - 95583	1
Glucose	14.74	[M+HCOO-]-	(-)	225	179	8	225	89	13	225	59	22	0.01 - 2.5	y = 473953x + 23292	0.99
Glycerol	21.10	[M+H]+	(+)	93	57	-11	93	75	-9	93	45	-14	0.001 - 10	y = 6E+06x + 121362	1
Glycolic acid	19.30	[M+HCOO-]-	(-)	121	75	9	121	47	13	121	45	14	0.01 - 2.5	y = 49705x + 3883.6	0.99
Glyoxylic acid	15.20	[M-H]-	(-)	73	45	10	73	29	11	73	29	11	0.08 - 2.5	y = 3727.5x + 2955.4	0.98
Inositol	15.27	[M+HCOO-]-	(-)	225	179	11	225	45	23	225	161	18	0.04 - 5	y = 61230x + 34912	0.99
Iso/Citric acid	13.29	[M-H]-	(-)	191	111	12	191	87	17	191	85	16	0.01 - 5	y = 9E+06x - 323210	1
Itaconic acid	20.27	[M+H]+	(+)	131	113	-12	131	85	-14	131	57	-19	0.001 - 2.5	y = 6E+06x + 63237	1
Lactose	12.86	[M+HCOO-]-	(-)	387	161	12	387	341	8	387	179	15	0.02 - 2.5	y = 1E+06x - 47967	1
Malic acid	15.35	[M-H]-	(-)	133	115	15	133	71	15	133	73	16	0.01 - 10	y = 1E+06x - 29988	1
Maltose	12.60	[M+HCOO-]-	(-)	387	161	12	387	341	8	387	179	15	0.16 - 20	y = 18807x + 74334	0.99

Mannitol	16.32	[M-H]-	(-)	181	89	15	181	101	14	181	71	21	0.01 - 1.25	y = 598007x - 6898.2	0.99
Mannose	15.50	[M+HCOO-]-	(-)	225	179	8	225	119	12	225	59	23	0.01 - 1.25	y = 2E+06x + 151810	0.98
Propionic acid	27.82	[M+H]+	(+)	75	57	-13	75	29	-16	75	27	-24	0.005 - 20	y = 6E+06x - 362268	1
Quinic acid	15.97	[M-H]-	(-)	191	85	21	191	93	22	191	127	18	0.01 - 10	y = 710721x + 89781	0.99
Rhamnose	16.32	[M-H]-	(-)	163	59	13	163	103	8	163	89	6	0.16 - 2.5	y = 20504x + 8738.4	0.98
Ribose	17.41	[M+HCOO-]-	(-)	195	149	10	195	89	12	195	45	23	0.02 - 10	y = 211084x + 28765	0.99
Sorbitol	16.50	[M-H]-	(-)	181	89	14	181	101	15	181	71	21	0.312 - 20	y = 129173x + 187056	0.99
Sorbose	15.02	[M-H]-	(-)	179	89	9	179	59	17	179	71	16	0.08 - 10	y = 107485x + 63268	0.98
Succinic acid	18.89	[M-H]-	(-)	117	100	23	117	73	16	117	99	14	0.04 - 2.5	y = 114364x - 93867	0.99
Sucrose	12.66	[M+Na]+	(+)	365	203	-23	365	185	-21	365	23	-40	0.001 - 1.25	y = 3E+07x + 525075	1
Tartaric acid	13.88	[M-H]-	(-)	149	87	13	149	73	16	149	103	13	0.02 - 20	y = 795107x + 43206	1
Trehalose	12.55	[M+HCOO-]-	(-)	387	341	13	387	179	19	387	89	26	0.001 - 0.312	y = 9E+06x + 85156	0.98
Xylitol	17.76	[M+H]+	(+)	153	107	-13	153	79	-16	153	61	-30	0.005 - 1.25	y = 1E+06x + 67560	0.98
Xylose	15.59	[M-H]-	(-)	195	89	12	195	149	10	195	59	21	0.04 - 20	y = 146820x + 36513	0.99
